# Salivary Duct Carcinoma in the External Auditory Canal Causing Ipsilateral Hearing Loss

**DOI:** 10.3390/diagnostics11081312

**Published:** 2021-07-21

**Authors:** Shih-Lung Chen, Chi-Ju Yeh, Kai-Chieh Chan

**Affiliations:** 1 Department of Otolaryngology & Head and Neck Surgery, Chang Gung Memorial Hospital, Linkou 333, Taiwan; rlong289@gmail.com; 2 School of Medicine, Chang Gung University, Taoyuan 333, Taiwan; t22259@cgmh.org.tw; 3 Department of Pathology, Chang Gung Memorial Hospital, Linkou 333, Taiwan; 4 Department of Otolaryngology, Chang Gung Memorial Hospital, Linkou 333, Taiwan

**Keywords:** salivary duct carcinoma, external auditory canal, hearing loss, androgen receptor, human epidermal growth factor receptor

## Abstract

**Background**: Salivary duct carcinoma (SDC) is a rare aggressive tumor. Most tumors are not confined to the salivary ducts; rather, they invade the major and minor salivary glands. Only a few case reports on such tumors in other primary sites have appeared. **Case presentation:** A 40-year-old male complained of right hearing loss (a common condition), but we made an extremely rare diagnosis of an SDC in the external auditory canal (EAC). EAC cancers are frequently misdiagnosed. In our patient, the otoscope revealed a smooth, bulging subcutaneous lesion with a non-epithelial defect suggestive of a benign lesion. However, an SDC of the EAC was confirmed through pathological and immunohistochemical analysis. **Conclusions:** We suggest detailed evaluation of even smooth EAC subcutaneous lesions to avoid erroneous diagnoses. To the best of our knowledge, this is the first report of SDC in the EAC.

## 1. Introduction

Salivary duct carcinoma (SDC) is an extremely rare but aggressive tumor originating from the ductal epithelial cells of the salivary glands [1]. SDC was first described in 1968 by Kleinsasser et al. [2]. SDC typically involves the major salivary glands, but approximately 10% of all SDCs are diagnosed in the minor salivary glands, palate, and oral cavity [3]. Other primary sites are extremely rare [1,3,4,5].

Herein, we report a common complaint of right hearing loss by a 40-year-old male, following which an extremely rare diagnosis of SDC in the external auditory canal (EAC) was made. To the best of our knowledge, this is the first such report.

## 2. Case Report

**Figure 1:**
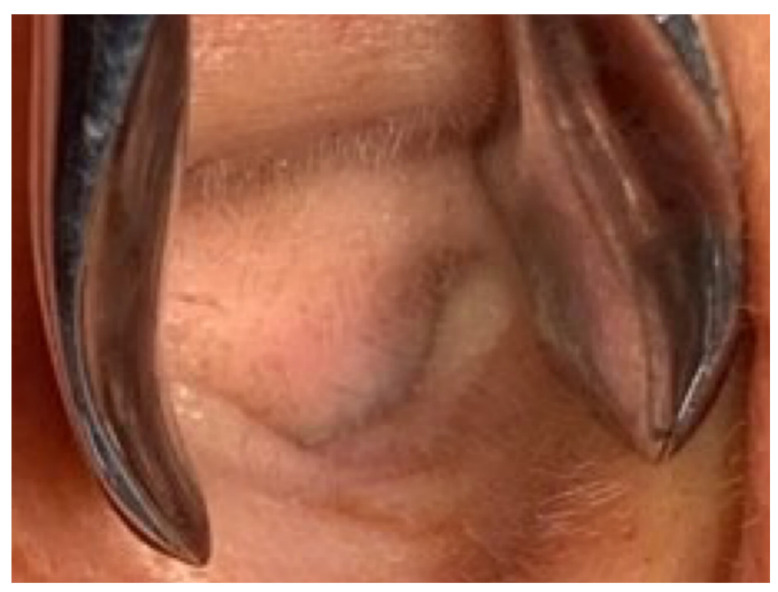
Otoscopy revealed a smooth, subcutaneous bulging mass that totally occluded the external auditory canal (EAC).

**Figure 2:**
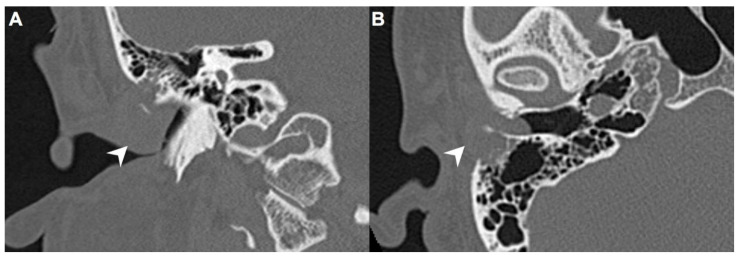
(**A**) On high-resolution computed tomography (HRCT), right EAC was occupied by a mass (arrow) on coronal view. (**B**) This submucosal soft tissue mass (arrow) was accompanied by partial mastoid bony destruction on axial view.

**Figure 3:**
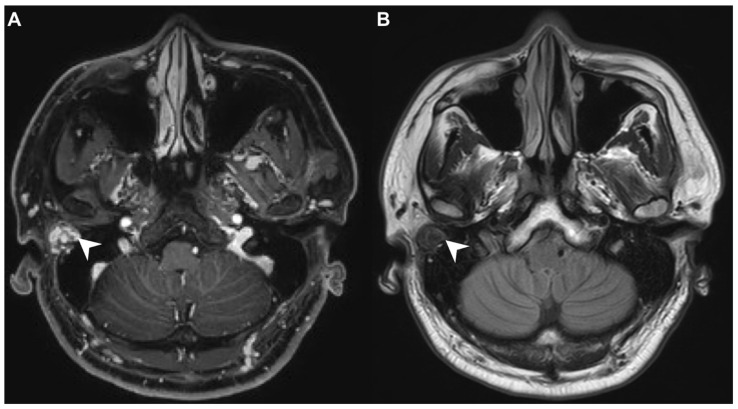
(**A**) On magnetic resonance imaging (MRI), the T1-contrast sequence revealed a heterogeneous hyperintense mass in the right EAC (arrow). (**B**) On MRI, the T2-sequence yielded a well-circumscribed isointense signal from the mass (arrow).

A 40-year-old male with hypertension was taking appropriate medication. He had suffered from right hearing impairment and aural fullness for two months. Examination with an otoscope revealed a smooth bulging mass with a non-epithelial defect on the right side that totally occluded the EAC (Figure 1). No otalgia, otorrhea, vertigo, or facial palsy was noted. There were no other lesions in the head-and-neck region. Audiological assessment revealed conductive hearing loss (pure-tone average) of 30 dB and an air-bone conduction gap of 20 dB on the right side, with preservation of bone conduction hearing. The laboratory data revealed a mildly elevated C-reactive protein level (7.44 mg/L; normal <5 mg/L) without leukocytosis (5400/µL; normal 3900–10,600/µL). As the EAC was occluded, high-resolution computed tomography (HRCT) was scheduled and revealed a submucosal soft tissue mass accompanied by partial mastoid bony destruction (Figure 2A,B). Magnetic resonance imaging (MRI) revealed a well-circumscribed mass in heterogeneous T1-weighted hyperintense (Figure 3A) and T2-weighted isointense (Figure 3B) images.

**Figure 4:**
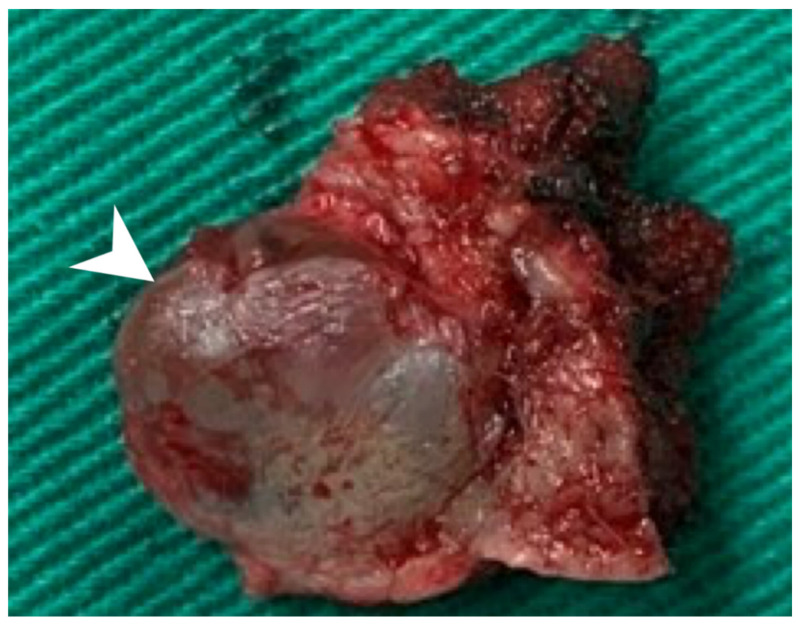
The 1.8 × 1.6 cm mass with safe margins (arrow).

**Figure 5:**
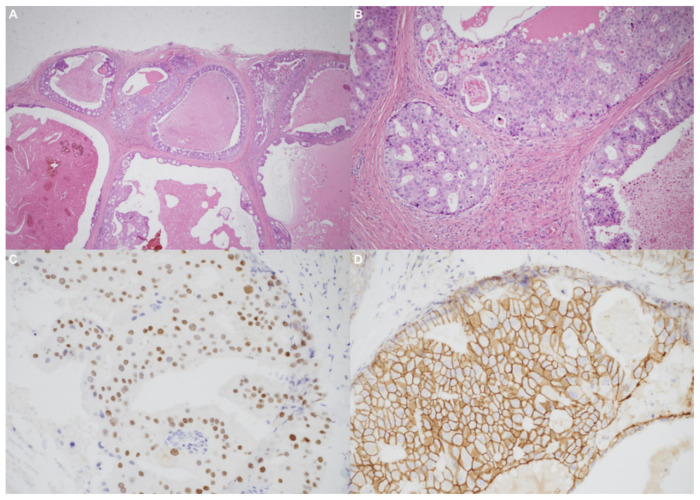
(**A**) The tumor resembled high-grade ductal carcinoma of the breast. Large ducts with comedo necrosis and Roman-bridge-like features are evident. Original magnification ×20. (**B**) A cribriform architectural pattern was observed. Original magnification ×200. (**C**) Immunohistochemical staining produced diffusely positive signals for the androgen receptor. (**D**) Significant human epidermal growth factor receptor 2 expression was noted.

Using an endaural approach, a 1.8 × 1.6 cm EAC tumor was surgically extirpated via canalplasty (Figure 4). A full-thickness skin graft from the posterior part of the auricle was used to cover the skin defect. Histopathological analysis revealed tumor cells in a cribriform pattern (Figure 5A,B). Positive immunohistochemical results were obtained for the androgen receptor (AR; Figure 5C), cytokeratin 7(CK7), G-A-T-A nucleotide-binding protein 3 (GATA3), gross cystic disease fluid protein 15 (GCDFP-15), and human epidermal growth factor receptor 2 (HER2; Figure 5D). The cells were negative for estrogen receptor (ER), progesterone receptor (PR), cytokeratin 20 (CK20), prostate-specific antigen (PSA), and calcium-binding protein soluble in a 100% saturated ammonium sulfate solution (S-100 protein). Therefore, the diagnosis was SDC in the right EAC. Concurrent chemoradiotherapy (CCRT) (featuring a cisplatin-based regimen) was initiated because, although the margin was tumor-free, the tumor came close to the margin. No residual tumor was evident on postoperative positron emission tomography. Further, no tumor recurrence was apparent during the 1-year follow-up.

## 3. Discussion

Carcinoma in the EAC is life-threatening and rare; the annual incidence is one per 1,000,000 population. The most common EAC cancer is squamous cell carcinoma [6]. SDC in the EAC presents as a rapidly enlarging firm mass, usually in the fifth and sixth decades of life. The male:female ratio is 3:1 [7].

Our patient initially presented with ipsilateral hearing loss and EAC bulging without crusting, otorrhea or facial palsy. A subcutaneous lesion lacking an epithelial defect (Figure 1) is not suggestive of a malignancy. Chen et al., reported that otalgia, otorrhea, facial palsy and vertigo were symptoms of EAC malignancies [8]. Ouaz et al., considered EAC cancers hard to diagnose unless the tumor presented as a fungating mass protruding from the EAC [6]. Zhang et al., concluded that EAC cancers are often misdiagnosed as otitis externa or otitis media [9]. Our patient complained only of hearing impairment. His age (40 years) differed from the typical age at which malignancies occur. There was no other head-and-neck lesion. CT and MRI both revealed an EAC tumor without any suspicious neck metastasis. Thus, it is possible to misdiagnose a malignant lesion as benign. Therefore, when managing patients with EAC lesions, clinicians should be aware of possible malignancies. We suggest advanced examination or biopsy of even seemingly harmless tumors.

In our patient, HRCT of the temporal bone revealed a well-demarcated, submucosal nodular lesion in the right EAC with tiny bony internal fragments (Figure 2A,B). MRI is indicated when HRCT shows a tumor involved with adjacent EAC soft tissues. In our patient, MRI revealed a well-circumscribed mass in the EAC that was hyperintense in the T1 (with contrast) sequence but T2-isointense (Figure 3A,B). Noda et al., and Pons et al., reported similar findings; T1-weighted contrast-enhanced MRI enabled strong tumor enhancement [10,11].

The fissure of Santorini allows the EAC to access the parotid gland [9]. However, neither physical examination nor imaging revealed any lesions in the salivary glands or salivary ducts. It is unknown whether the SDC invaded the EAC via the fissure or arose from the EAC epithelium.

Primary EAC cancers are rare; accurate and early histopathological diagnosis is thus essential [12]. Histologically, SDC resembles invasive ductal carcinoma of the breast [10]. The tumor cell pattern is cribriform and comedo necrosis is microscopically apparent [13]. Roman bridge-like structures are common to ductal carcinoma of the breast as well as SDC, and most SDCs express the AR and HER2 [1].

No consensus therapy has yet emerged, as very few SDC patients have been described. In general, EAC cancers undergo radical excision (if they are resectable) with concurrent CCRT [13].

Zhang et al., considered it difficult to obtain a clear margin when the tumor involves surrounding vital structures [9]. Therefore, CCRT is suggested to improve locoregional control [14].

Despite such aggressive treatment, the five year disease-specific and disease-free survival rates are less than 50% [1].

The overall SDC response rates to platinum (carboplatin) chemotherapy and taxane (paclitaxel or docetaxel) are also less than 50% [13]. Di et al., surmised that some patients had undergone inadequate initial surgery [15]. Incomplete surgery (associated with a residual tumor) would render a poor outcome. In such a situation, postoperative radiotherapy (RT) could improve locoregional control but could not counter distant metastases [15]. In patients with lymph node metastases, neck dissection and adjuvant postoperative RT are indicated [16].

Therapies targeting the AR and HER2 are under development. Currently, androgen-deprivation therapy employs AR blockers (bicalutamide) and luteinizing hormone-releasing hormone analogs (leuprolide) [13]. As trastuzumab was useful in patients with HER2-positive breast cancers, a therapeutic trial of trastuzumab in patients with HER2-positive SDCs has been suggested [17].

SDC has a poor prognosis as it is associated with a high recurrence rate and distant metastasis despite radical surgery and CCRT [1]. Adverse prognostic factors include facial nerve involvement, vascular or perineural invasion, and advanced neck lymph node metastasis [18]. The five year disease-free survival rate was reported to be 36% [19]. Our patient lacks any signs of recurrence at the time of writing, but we have of course scheduled long-term follow-up.

## 4. Conclusions

Single-side hearing loss is common, but we diagnosed an extremely rare SDC in the EAC. As SDC is aggressive and associated with poor prognosis, differential diagnosis of an EAC mass is essential. EAC cancers are frequently misdiagnosed. We suggest that detailed evaluation of even smooth subcutaneous lesions in the EAC is appropriate.

## Data Availability

All data generated or analyzed during this study are included in this published article.

## Abbreviations

EAC	external auditory canal
AR	androgen receptor
HER2	human epidermal growth factor receptor 2
SDC	salivary duct carcinoma.
